# Bidirectional two-sample Mendelian randomization analysis reveals a causal effect of interleukin-18 levels on postherpetic neuralgia risk

**DOI:** 10.3389/fimmu.2023.1183378

**Published:** 2023-05-25

**Authors:** Xiao Liang, Yuchao Fan

**Affiliations:** ^1^ Department of Anesthesiology, West China Hospital, Sichuan University, Chengdu, Sichuan, China; ^2^ Department of Anesthesiology, Sichuan Clinical Research Center for Cancer, Sichuan Cancer Hospital & Institute, Sichuan Cancer Center, Affiliated Cancer Hospital of University of Electronic Science and Technology of China, Chengdu, China

**Keywords:** postherpetic neuralgia, interleukin-18, bidirectional Mendelian randomization, genome-wide association study, herpes zoster

## Abstract

**Background:**

Postherpetic neuralgia (PHN) is a debilitating complication of herpes zoster, characterized by persistent neuropathic pain that significantly impairs patients’ quality of life. Identifying factors that determine PHN susceptibility is crucial for its management. Interleukin-18 (IL-18), a pro-inflammatory cytokine implicated in chronic pain, may play a critical role in PHN development.

**Methods:**

In this study, we conducted bidirectional two-sample Mendelian randomization (MR) analyses to assess genetic relationships and potential causal associations between IL-18 protein levels increasing and PHN risk, utilizing genome-wide association study (GWAS) datasets on these traits. Two IL-18 datasets obtained from the EMBL’s European Bioinformatics Institute database which contained 21,758 individuals with 13,102,515 SNPs and Complete GWAS summary data on IL-18 protein levels which contained 3,394 individuals with 5,270,646 SNPs. The PHN dataset obtained from FinnGen biobank had 195,191 individuals with 16,380,406 SNPs.

**Results:**

Our findings from two different datasets of IL-18 protein levels suggest a correlation between genetically predicted elevations in IL-18 protein levels and an increased susceptibility to PHN.(IVW, OR and 95% CI: 2.26, 1.07 to 4.78; p = 0.03 and 2.15, 1.10 to 4.19; p =0.03, respectively), potentially indicating a causal effect of IL-18 protein levels increasing on PHN risk. However, we did not detect any causal effect of genetic liability to PHN risk on IL-18 protein levels.

**Conclusion:**

These findings suggest new insights into identifying IL-18 protein levels increasing at risk of developing PHN and may aid in the development of novel prevention and treatment approaches for PHN.

## Introduction

Herpes zoster (HZ), caused by the varicella-zoster virus (VZV), is a viral infection that can affect individuals of all ages but is more common in the elderly and those with weakened immune systems. The prevalence of HZ increases with advancing age. Typical symptoms of HZ include localized pain, burning, or tingling in specific dermatomes such as the head, neck, arms, trunk (including chest and back), and legs, often unilateral. The incidence of HZ increases with age. The characteristic symptoms of HZ include a painful, burning, or tingling sensation in a specific body area, often on one side of the trunk. The affected area may also be itchy, painful, and sensitive to touch. A rash of fluid-filled blisters usually appears in the same area within a few days. Accompanied by fever, headache, fatigue, and general malaise, the rash typically lasts for 2-4 weeks (1).

Postherpetic neuralgia (PHN) is a common and debilitating complication of HZ. This torturous neuropathic pain is characterized by persistent pain in the affected dermatomes, leading to significant impairment in the patient’s quality of life, including sleep disturbances, depression, anxiety, and decreased physical functioning (2). PHN is associated with increased personal and societal medical costs, loss of productivity, and a significant decrease in the quality of life of patients and their caregivers (3).

PHN results from sensory nerve damage caused by the HZ virus. The likelihood of developing PHN is linked to age, initial infection severity, and other medical conditions. Early recognition and prompt application of antiviral medications can reduce the risk of PHN and improve patient prognosis (4). Managing PHN requires a multidisciplinary approach, including physical therapy, pharmacotherapy, nerve blocks, or destructive therapy such as the using Adriamycin for spinal dorsal root ganglion destruction. However, each treatment has adverse effects and may not provide complete pain relief or be effective. Immunization against HZ prevents PHN by reducing the incidence and severity of the disease (5). Vaccine adoption, however, remains low. Zoster vaccine live (ZOSTAVAX) and RZV (SHINGRIX) have obtained licensure or distribution in a total of 62 nations across the globe. Despite being granted regulatory approval in each of the 28 constituent countries of the European Union, only 9 of them have explicitly incorporated recommendations pertaining to HZ vaccination into their respective national vaccination strategies (6). Early and effective PHN treatment is essential for alleviating long-term patient suffering and reducing associated medical expenditures. Differences in physiological factors, such as immune function, may determine PHN susceptibility, and understanding these factors can improve prevention and treatment. The elucidation of such factors is crucial to advance PHN management.

Interleukin-18 (IL-18) is a pro-inflammatory cytokine involved in the immune response to infection and inflammation, produced by various cells, including macrophages, dendritic cells, and epithelial cells. IL-18 acts on immune cells such as T cells and natural killer cells, regulating the immune response by promoting interferon-gamma and other cytokine production (7). Dysregulation of IL-18 is associated with several inflammatory and autoimmune diseases, including inflammatory bowel disease, rheumatoid arthritis, and multiple sclerosis (8). Recent studies suggest that there is an association between IL-18 and chronic pain, and this cytokine has been implicated in the pathogenesis of a variety of pain conditions, including neuropathic pain, osteoarthritic pain, and cancer pain (9–11). In particular, in the context of neuropathic pain, an increase in IL-18 levels has been observed across a range of models including Compression of Dorsal Root Ganglion (12), Spinal Cord Injury (13), and Chronic Constriction Injury of the sciatic nerve (14–17). Moreover, higher IL-18 levels were reported in patients with HZ compared to controls (18). Additionally, IL-18 has been proposed to play a pivotal role in the modulation of nociceptive transmission in neuropathic pain (19–22), and attenuating IL-18 signaling or reducing its levels may alleviate neuropathic pain symptoms (19, 23–25). Thus, we hypothesize that IL-18 protein levels may play a critical role in PHN development. In this study, we aim to explore the causal relationship between IL-18 protein levels (ebi-a-GCST90012024 and prot-b-21) and PHN (finn-b-G6_POSTZOST) using a bidirectional, two-sample Mendelian randomization (MR) analysis with the latest genome-wide association study (GWAS) database. The findings from this study may provide evidence for effective PHN prevention and treatment strategies, identify high-risk groups for PHN, facilitate the deployment of preventive vaccines, and generate ideas for developing new treatments for PHN.

## Methods

### Study design

**Figure 1:**
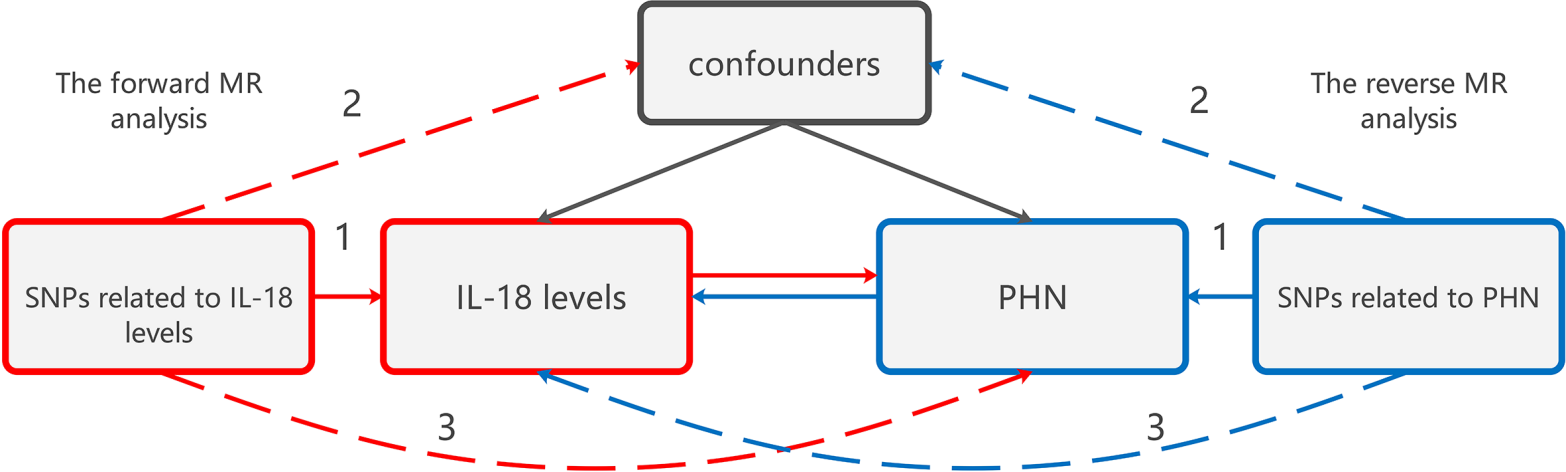
The design of bidirectional MR study examining the relationship between IL-18 protein levels increasing and PHN. The red analysis investigates the causal effect of IL-18 protein levels increasing as the exposure on PHN as the outcome, while the blue analysis examines the reverse association. To serve as a valid instrument, the genetic variant used in the study must meet three criteria: (1) The genetic variant is associated with the exposure. (2) The genetic variant is not associated with any confounders of the exposure-outcome association. (3) The genetic variant does not affect the outcome, except through association with exposure. MR, Mendelian randomization;PHN, Postherpetic neuralgia; IL, Interleukin.

This study utilized bidirectional two-sample MR analyses based on summary statistics from a GWAS to examine the bidirectional association between IL-18 protein levels increasing and PHN. The forward MR analyses investigated IL-18 protein levels increasing as the exposure and PHN as the outcome, while the reverse MR analyses explored PHN as the exposure and IL-18 protein levels as the outcome (**Figure 1**). As publicly available summary statistics were used, no ethical approval was required.

The genetic variation was utilized to assess the causal effect of exposure on outcome. The fundamental conditions for genetic variation to satisfy the instrumental variables (IVs) in this study are as follows: (1) The genetic variant is associated with the exposure. (2) The genetic variant is not associated with any confounders of the exposure-outcome association. (3) The genetic variant does not affect the outcome, except possibly through association with exposure.

### Data source

This study employed genetic associations derived from two independent GWAS datasets as either exposure or outcome, one for IL-18 protein levels and the other for PHN. Two separate GWAS datasets of IL-18 protein levels were used for MR Analysis to enhance the power of the study. One GWAS data for IL-18 protein levels was obtained from ebi-a-GCST90012024 (26), which obtained from the EMBL’s European Bioinformatics Institute Catalog of human genome-wide association studies and included a sample size of 21,758 individuals of European descent and contained 13,102,515 SNPs. An additional GWAS dataset (prot-b-21) was utilized to reinforce the result. This dataset is based on Complete GWAS summary data on protein levels (27), which involved a sample size of 3,394 individuals of European ancestry and encompassed 5,270,646 SNPs. The PHN dataset was obtained from finn-b-G6_POSTZOST (FinnGen biobank analysis round 5), also from a European population, with a case size of 144 and a control group of 195,047 individuals and containing 16,380,406 SNPs. There was no overlap in samples between the three datasets.

### Selection of instrumental variables

The IVs used in this study were selected based on the following criteria: (1) significant genome-wide association (P< 5e-8) with the exposure, or P-value < 1e-5 if no relevant SNPs were identified, and minor allele frequency (MAF) in the outcome > 0.01; (2) linkage disequilibrium (LD) r2 < 0.001 within a 10,000 kb distance. SNPs associated with confounders or outcomes in the Phenoscanner (http://www.phenoscanner.medschl.cam.ac.uk/) were excluded. We subsequently harmonized the combined SNP-IL-18 and SNP-PHN statistics to ensure allelic consistency between IL-18 protein levels and PHN.

The proportion of variance explained by individual SNPs was calculated using R2 = 2 × β2 × EAF × (1 − EAF)/(2 × β2 × EAF × (1 − EAF) + 2 × SE2 × N × EAF × (1 − EAF)). The F-statistic was computed using the formula F = (N − k − 1)/k ×R2/(1 − R2), where N represents the number of samples exposed to the GWAS study, k represents the number of IVs, and R2 is the extent to which IVs explain the exposure. When the F-statistic is less than 10, the genetic variants used are considered weak instrumental variables, which may bias the results somewhat. Therefore, caution is necessary when interpreting the results (28).

We performed five MR methods to assess the causal association between IL-18 protein levels and PHN, with the IVW method as the primary MR analysis and MR Egger, weighted median, simple mode, and weighted mode methods as supplementary analyses. A causal effect of exposure on the increase in the risk of outcome was considered significant if p < 0.05. Effect estimates from MR analyses were reported as odds ratios (ORs) with corresponding 95% confidence intervals (CIs). Heterogeneity was assessed using the mr_egger and IVW in Cochran’s Q statistic, with a p-value greater than 0.05 indicating no heterogeneity. In cases where heterogeneity was present, we excluded or estimated SNPs directly using a random effects model (29). We tested the pleiotropy using the intercept p-value obtained from the MR Egger regression and global test p-value of MR-PRESSO, with p > 0.05 indicating no potential pleiotropy of IVs (30). Furthermore, we conducted a “leave-one-out” sensitivity analysis to demonstrate that the causal effect of exposure on outcome was not influenced by individual SNPs. We used R software (version 4.2.2) and R Package “TwoSampleMR” and “MRPRESSO” to perform all statistical analyses in the MR analysis.

## Results

### The causal effect of IL-18 protein levels on PHN

Using the ebi-a-GCST90012024 dataset, eight SNPs were extracted from the forward MR analysis process to investigate the causal effect of IL-18 protein levels on PHN. No SNPs were excluded following clumping. One SNP (rs10424405) was removed when extracting outcome data due to its failure to meet inclusion criteria. The remaining seven SNPs demonstrated a significant association with elevated IL-18 protein levels. Meanwhile, three SNPs meeting the inclusion criteria of IVs were selected from the prot-b-21 dataset, and none of them were excluded during the clumping process. These SNPs were also successfully harmonized with the outcome data later on. These SNPs were not associated with PHN or related confounders in Phenoscanner and were therefore treated as IVs. The F-values for all SNPs were greater than 10, indicating that they were not weak instruments (**Table 1**).

**Table 1:** Instrumental variants (IVs) details in MR analyses.

Dataset		IVs	Exposure			outcome			effect_allele.exposure	other_allele.exposure	effect_allele.outcome	other_allele.outcome	chr	pos	F	R2
			Beta	SE	p Value	Beta	SE	p Value								
**Exposure**	**Outcome**	**The forward MR analyses**														
ebi-a-GCST90012024	finn-b-G6_POSTZOST	rs17229943	0.182	0.028	6.41E-11	0.018	0.197	0.929	C	A	C	A	5	68682536	14.197	0.0019541
		rs385076	0.185	0.012	3.19E-55	0.249	0.123	0.044	C	T	C	T	2	32489851	81.476	0.0111111
		rs4606077	0.088	0.012	4.57E-13	0.142	0.130	0.275	C	T	C	T	8	144656754	17.379	0.002391
		rs547351	-0.080	0.014	8.01E-09	0.011	0.143	0.937	A	T	A	T	11	104883858	11.144	0.0015345
		rs5744249	-0.218	0.012	3.74E-74	-0.177	0.138	0.200	C	A	C	A	11	112025306	110.292	0.014982
		rs916964	-0.074	0.013	6.41E-09	0.087	0.149	0.560	G	C	G	C	7	26146063	11.254	0.0015496
		rs9867857	0.060	0.010	5.12E-09	0.019	0.119	0.875	T	C	T	C	3	156491160	11.385	0.0015675
prot-b-21	finn-b-G6_POSTZOST	rs693918	0.191	0.029	2.38E-11	0.305	0.119	0.010	G	A	G	A	2	31868877	44.961	0.013073879
		rs75649625	-0.289	0.030	1.46E-21	-0.113	0.130	0.387	A	G	A	G	5	112052194	31.680	0.009247863
		rs76003549	1.220	0.217	1.95E-08	0.817	0.676	0.227	A	C	A	C	11	140438557	92.441	0.026514397
**Exposure**	**Outcome**	**The reverse MR analyses**														
finn-b-G6_POSTZOST	ebi-a-GCST90012024	rs10437632	1.867	0.401	3.20E-06	-0.040	0.030	0.187	T	G	T	G	11	37692027	20.501	0.001117
		rs10974880	0.606	0.122	7.33E-07	-0.018	0.011	0.120	A	G	A	G	9	4949635	20.953	0.03696
		rs112340368	1.849	0.379	1.07E-06	0.011	0.032	0.733	T	C	T	C	11	10680571	19.841	0.001571
		rs114851674	1.519	0.334	5.34E-06	-0.012	0.029	0.680	A	G	A	G	5	157267843	20.704	0.000502
		rs115160454	1.418	0.310	4.71 E-05	0.064	0.043	0.131	A	G	A	G	3	27557667	20.091	0.000758
		rs116775850	4.624	1.038	8.42E-06	0.132	0.076	0.082	T	C	T	C	5	119302316	21.765	5.41E-05
		rs117847964	1.972	0.438	6.70E-05	0.032	0.039	0.407	C	A	C	A	21	28890333	19.758	3.21E-05
		rs140949248	3.333	0.736	5.96E-06	-0.036	0.076	0.638	G	A	G	A	3	326807	24.520	0.000131
		rs149106337	2.536	0.560	5.84E-06	0.043	0.065	0.503	G	A	G	A	15	26664454	20.213	0.000395
		rs180673827	2.969	0.608	1.04E-06	-0.035	0.062	0.574	C	A	C	A	22	45039075	27.484	1.34E-05
		rs192457298	3.272	0.728	6.94E-06	0.072	0.059	0.219	A	G	A	G	10	70140028	23.718	0.000134
		rs2745867	0.708	0.158	7.75E-05	-0.001	0.014	0.927	A	C	A	C	20	17851119	23.799	0.000212
		rs2952725	0.574	0.123	3.09E-05	0.009	0.012	0.456	A	G	A	G	7	9602259	21.692	0.153329
		rs55868313	1.147	0.258	8.81E-06	0.038	0.022	0.090	T	C	T	C	9	95349619	19.576	0.001196
		rs72879582	2.707	0.556	1.12E-06	-0.066	0.032	0.037	A	T	A	T	11	30702138	20.542	0.000356
		rs75575295	2.493	0.524	1.96E-06	-0.045	0.035	0.200	G	A	G	A	18	45425597	20.188	2.55E-05
		rs76412908	3.618	0.807	7.38E-06	-0.043	0.059	0.460	C	T	C	T	6	93324967	22.630	8.07E-05
		rs77570959	4.438	0.847	1.583E-07	-0.165	0.076	0.029	G	A	G	A	11	73735375	20.001	8.01E-05
		rs9549848	2.129	0.481	9.68E-06	0.065	0.047	0.171	A	C	A	C	13	112773394	20.280	0.000636
		rs9616188	2.044	0.456	7.18E-06	0.003	0.027	0.926	T	C	T	C	22	46515834	23.844	5.74E-05
		rs9945240	1.299	0.289	7.03E-06	0.029	0.016	0.067	G	A	G	A	18	44139893	20.140	0.001855
finn-b-G6_POSTZOST	prot-b-21	rs10974880	0.606	0.122	7.33E-07	0.006	0.025	0.814	A	G	A	G	9	4949635	20.953	0.03696
		rs114851674	1.519	0.334	5.35E-06	-0.151	0.074	0.041	A	G	A	G	5	157267843	20.704	0.000502
		rs115160454	1.418	0.310	4.71E-06	0.077	0.103	0.452	A	G	A	G	3	27557667	20.091	0.000758
		rs117847964	1.972	0.438	6.70E-06	0.072	0.122	0.557	C	A	C	A	21	28890333	19.758	3.21E-05
		rs2745867	0.708	0.158	7.75E-06	0.020	0.035	0.565	A	C	A	C	20	17851119	23.799	0.000212
		rs2952725	0.574	0.123	3.09E-06	-0.022	0.038	0.559	A	G	A	G	7	9602259	21.692	0.153329
		rs55868313	1.147	0.258	8.81E-06	0.053	0.098	0.592	T	C	T	C	9	95349619	19.576	0.001196
		rs9945240	1.299	0.289	7.03E-06	0.051	0.045	0.257	G	A	G	A	18	44139893	20.140	0.001855

IVs, instrumental variants; MR, Mendelian randomization; SE, standard error; SNP, single nucleotide polymorphism.

**Figure 2:**
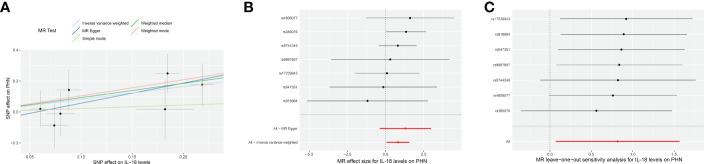
The forward MR using IL-18 protein levels (ebi-a-GCST90012024) increasing as exposure and PHN risk as outcome. **(A)** Scatter plot in the MR analysis of IL-18 protein levels increasing on PHN risk. The plot shows individual MR estimates, indicating that as the effect of individual SNPs on IL-18 protein levels increasing, so does the promotion of PHN occurrence by individual SNPs. The x-axis indicates the SNP effect and standard error on IL-18 levels for each of the seven SNPs, while the y-axis shows the SNP effect and standard error on PHN. The plot includes the regression line for mr_egger, weighted median, IVW, simple mode, and weighted mode. **(B)** Display of the forest plot for the single SNP analysis of IL-18 protein levels increasing on PHN risk. The x-axis shows the MR effect size for IL-18 protein levels increasing on PHN, while the y-axis illustrates the analysis for each of the SNPs. The dot and bar indicate the causal estimate and 95% CI of the association between IL-18 protein levels increasing and PHN risk. **(C)** Presentation of the leave-one-out sensitivity analysis for the effect of IL-18 protein levels increasing SNPs on PHN risk in the context of MR. The dot and bar indicate the estimate and 95% CI when a specific SNP is removed. IV, instrumental variant; IVW, inverse variance weighted; MR, Mendelian randomization; SE, standard error; SNP, single‐nucleotide polymorphism; PHN, Postherpetic neuralgia; IL, Interleukin.

**Figure 3:**
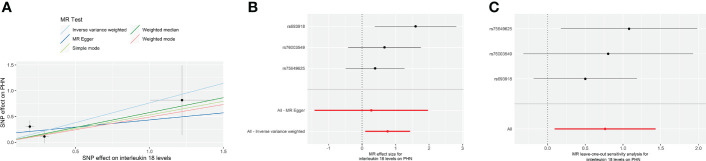
The forward MR using IL-18 protein levels (prot-b-21) increasing as exposure and PHN risk as outcome. **(A)** Scatter plot in the MR analysis of IL-18 protein levels increasing on PHN risk. The plot shows individual MR estimates, indicating that as the effect of individual SNPs on IL-18 protein levels increasing, so does the promotion of PHN occurrence by individual SNPs. The x-axis indicates the SNP effect and standard error on IL-18 levels for each of the seven SNPs, while the y-axis shows the SNP effect and standard error on PHN. The plot includes the regression line for mr_egger, weighted median, IVW, simple mode, and weighted mode. **(B)** Display of the forest plot for the single SNP analysis of IL-18 protein levels increasing on PHN risk. The x-axis shows the MR effect size for IL-18 protein levels increasing on PHN, while the y-axis illustrates the analysis for each of the SNPs. The dot and bar indicate the causal estimate and 95% CI of the association between IL-18 protein levels increasing and PHN risk. **(C)** Presentation of the leave-one-out sensitivity analysis for the effect of IL-18 protein levels increasing SNPs on PHN risk in the context of MR. The dot and bar indicate the estimate and 95% CI when a specific SNP is removed. IV, instrumental variant; IVW, inverse variance weighted; MR, Mendelian randomization; SE, standard error; SNP, single‐nucleotide polymorphism; PHN, Postherpetic neuralgia; IL, Interleukin.

The results from the IVW models showed that genetically predicted increases in IL-18 protein levels were associated with an increased risk of PHN (ebi-a-GCST90012024 and prot-b-21 OR and 95% CI: 2.26, 1.07 to 4.78; p = 0.03 and 2.15, 1.10 to 4.19; p =0.03, respectively) (**Table 2**, **Figures 2A, B**, **3A, B**). However, no significant association was observed in other models. The MR Egger and IVW in Cochran’s Q test did not show any significant heterogeneity among the IVs in the PHN GWAS (**Table 3**, ebi-a-GCST90012024 and prot-b-21, MR Egger p = 0.807, IVW p = 0.846 and MR Egger p = 0.181, IVW p = 0.283, respectively). Additionally, the MR Egger intercept p-value and global test p-value of MR-PRESSO results indicated no potential pleiotropy (**Table 3**, ebi-a-GCST90012024 and prot-b-21, MR Egger p = 0.555, MR-PRESSO p = 0.886 and MR Egger p = 0.683, MR-PRESSO p = 0.247, respectively), suggesting that there was no pleiotropy in these IVs.

**Table 2:** MR analysis of exposures with outcomes.

Datasets			β	se	p	OR	95%CI(OR)
Exposure	Outcome	The forward MR					
ebi-a-GCST90012024	finn-b-G6_POSTZOST	MR Egger	1.301	0.855	0.188	3.675	0.686-19.672
		Weighted median	0.923	0.451	0.040	2.517	1.039-6.098
		Inverse variance weighted	0.817	0.381	0.032	2.265	1.072-4.787
		Simple mode	0.222	0.645	0.742	1.248	0.352-4.429
		Weighted mode	1.039	0.486	0.076	2.828	1.090-7.337
prot-b-21	finn-b-G6_POSTZOST	MR Egger	0.276	0.865	0.804	1.317	0.242-7.168
		Weighted median	0.578	0.364	0.112	1.781	0.873-3.632
		Inverse variance weighted	0.763	0.342	0.026	2.146	1.098-4.193
		Simple mode	0.530	0.491	0.393	1.700	0.649-4.452
		Weighted mode	0.489	0.403	0.349	1.631	0.740-3.592
Exposure	Outcome	The reverse MR					
finn-b-G6_POSTZOST	ebi-a-GCST90012024	MR Egger	-0.003	0.008	0.672	0.996	0.979-1.013
		Weighted median	-0.001	0.006	0.853	0.998	0.987-1.010
		Inverse variance weighted	-0.000	0.004	0.938	0.999	0.990-1.009
		Simple mode	-0.011	0.014	0.453	0.989	0.961-1.017
		Weighted mode	-0.013	0.012	0.274	0.986	0.962-1.010
finn-b-G6_POSTZOST	prot-b-21	MR Egger	0.010	0.051	0.848	1.010	0.914-1.116
		Weighted median	0.031	0.024	0.197	1.031	0.984-1.081
		Inverse variance weighted	0.008	0.018	0.655	1.008	0.973-1.045
		Simple mode	0.039	0.032	0.270	1.040	0.976-1.108
		Weighted mode	0.035	0.028	0.248	1.036	0.981-1.093

MR, Mendelian randomization; SE, standard error; OR, odds ratio; CI, confidence intervals.

**Table 3:** Heterogeneity and pleiotropy analyses.

	Exposure	Outcome	Heterogeneity test				Pleiotropy test	
			IVW Q	p	MR‐Egger Q	p	MR‐Egger p	PRESSO p
The forward MR analyses	ebi-a-GCST90012024	finn-b-G6_POSTZOST	2.693	0.846	2.293	0.807	0.555	0.886
	prot-b-21	finn-b-G6_POSTZOST	2.521	0.283	1.789	0.181	0.638	0.247
The reverse MR analyses	finn-b-G6_POSTZOST	ebi-a-GCST90012024	32.837	0.035	32.466	0.027	0.646	0.026
	finn-b-G6_POSTZOST	prot-b-21	7.208	0.408	7.206	0.302	0.966	0.404

IVW, inverse variance weighted; MR, Mendelian randomization; PHN, Postherpetic neuralgia; IL, Interleukin.

All analyses supported an effect of each of the IL-18 protein levels increasing SNPs on PHN. MR exclusion sensitivity analysis revealed that removing any specific SNP among IL-18 protein levels SNPs did not affect the results (**Figures 2C**, **3C**). Thus, all selected IL-18 protein levels genetic IVs should be considered effective for this MR analysis.

### The reverse MR

Subsequently, we conducted a reverse MR analysis to investigate the causal relationship between PHN and IL-18 protein levels. Due to the lack of SNPs in the GWAS of PHN at a significance threshold of p < 5e-8, we obtained 21 independent genetic IVs from the GWAS of PHN using a less stringent threshold of p < 1e-5. Then, we extracted 21 PHN-associated genetic variants from the ebi-a-GCST90012024 dataset and 8 PHN-associated genetic variants from the prot-b-21 dataset (**Table 1**). MR-PRESSO detected significant pleiotropy in the 21 independent IVs from the GWAS of PHN, but no outlier was identified. Conversely, no significant pleiotropy was found from MR-Egger intercept p-value (**Table 3**). Both MR Egger and IVW revealed significant heterogeneity among the 21 independent IVs in Cochran’s Q statistics (**Table 3**). However, no significant pleiotropy or heterogeneity was found in 8 PHN-associated genetic variants from the prot-b-21 dataset. Therefore, we used the IVW method with random effects model as our primary statistical approach for ebi-a-GCST90012024 detaset and fix effects model for prot-b-21 dataset. The results of the IVW analysis suggested that there may be no causal effect of PHN on IL-18 protein levels (**Table 2**, **Supplementary Figures 1A**, **2A**). Moreover, the leave-one-out sensitivity analysis indicated that each SNP was heterogeneous with other SNPs (**Supplementary Figures 1B**, **2B**).

## Discussion

In the present study, we explored the causal relationship between IL-18 protein levels increasing and PHN risk using a bidirectional, two-sample MR method. To the best of our knowledge, this is the first MR study on PHN. In the forward MR analysis (IL-18 protein levels in PHN), we obtained non-significant heterogeneity and pleiotropy for the IVs. Additionally, we used PhenoScanner to identify SNPs with a high risk of pleiotropy as far as possible, indicating that the IVs in this study are valid. The IVs all had F values greater than 10, indicating sufficient strength. In addition, the exposure and sample datasets of this two-sample MR analysis were independent of each other, making the results more reliable and more extensive. The results of the forward MR analysis from two independent GWAS datasets on IL-18 protein levels suggest a potential causal relationship between elevated IL-18 protein levels and the incidence of PHN. In contrast, the inverse Mendelian randomization analysis utilizing two independent GWAS datasets on IL-18 protein levels as outcomes with either a random or fixed effects model of IVW method suggests no causal association between PHN and IL-18 protein levels. And no potential pleiotropy was found in inverse MR analysis. Compared with traditional observational studies, causal estimates derived from MR analysis can avoid reverse causality and multiple confounding biases (31). Also, applying integrated GWASs data for MR analysis can improve the accuracy of the estimates (32). Finally, it is noteworthy that we employed two distinct GWAS datasets for IL-18 protein levels to mutually validate each other, thereby augmenting the robustness of our findings.

Different from conventional MR analysis, bidirectional MR analysis is employed in this study to establish a causal relationship between elevated IL-18 levels and the development of PHN, while ruling out any reverse causality where the development of PHN leads to increased IL-18 levels as observed in pain studies involving neuropathic pain (9–11, 33–37). The conventional MR analysis assumes a unidirectional causal pathway between the exposure variable (e.g., genetic variant) and the outcome variable (e.g., disease risk). However, in some instances, there may exist a reverse causation scenario where the outcome variable also influences the exposure variable (32, 38). Bidirectional MR analysis takes into account the possibility of reverse causation by estimating causal effects in both directions: from the exposure variable to the outcome variable and from the outcome variable to the exposure variable (31, 39, 40). By comparing the results of both analyses, we can ascertain whether a causal relationship exists between the exposure and outcome variables or if reverse causation is at play. Therefore, utilizing bidirectional MR analysis can provide a more comprehensive and precise evaluation of the causal association between the exposure variable and outcome variable.

HZ is a prevalent viral disease caused by the reactivation of the varicella-zoster virus (VZV) in latently infected neurons. VZV can spread retrogradely through axons of cutaneous lesions or latent along the entire nerve axis to peripheral neurons, dorsal root ganglia, cranial nerve ganglia, and autonomic ganglia (41). HZ typically presents with a burning sensation or pain in the skin, accompanied by headache, general discomfort, and photophobia, two days before the outbreak. During the acute outbreak, severe skin pain, nociceptive hypersensitivity, and allodynia are common (1). The vast majority of individuals have been exposed to the VZV. Antibodies against the virus were detected in 97.3% of individuals aged between 20 and 39 years, whereas primary VZV infection was observed in 99.2% of individuals over the age of 40 (42). HZ occurs in approximately one-third of the populations who have been exposed to the VZV, and its likelihood of occurrence increases with age. Advanced age and immunosuppression are high-risk factors for the development of HZ (43). PHN is a common complication in 5-30% of patients with shingles, characterized by pain that persists for more than 3 months after an HZ attack. Patients with PHN experience pain, sensory disturbances, and sensory abnormalities, and in severe cases, the pain may be disabling and even life-threatening (44). PHN can cause significant physical, psychological, functional, and social impairment, making it the most debilitating sequelae of HZ (45).

The pathogenesis of PHN is multifaceted and intricate. Several factors, such as nerve damage, immune activation, and genetic predisposition, can all contribute to the development and persistence of chronic pain in this condition. VZV primarily infects sensory nerve fibers, resulting in inflammation and nerve cell damage. This damage can render the nerves hypersensitive, causing them to transmit pain signals more easily, even after the virus has cleared from the body (46–48). Another possible mechanism underlying PHN is immune activation, whereby the immune system produces cytokines and other inflammatory molecules that can damage nerve cells and lead to persistent pain. Furthermore, chronic inflammation can alter the way pain signals are processed in the central nervous system, thereby allowing pain to persist even after the initial injury has healed (47, 49, 50). Lastly, genetic factors may also influence the development of PHN. Several studies have identified specific genetic variants that are associated with an increased risk of developing PHN, suggesting a genetic predisposition to the disorder (51–53).

IL-18, a pro-inflammatory cytokine, is a pivotal regulator of immune responses and inflammation (8). Its association with chronic pain, including neuropathic pain, osteoarthritic pain, and cancer pain, has been extensively studied (9–11). Produced by macrophages, monocytes, and dendritic cells, IL-18 stimulates toll-like receptor and nuclear factor kb transcription, thereby promoting inflammation and increasing pain sensitivity (54, 55). Moreover, IL-18 exerts its pain-promoting effects by modulating pain-signaling pathways in the spinal cord and brain. By interacting with sensory neurons via the IL-18 receptor (IL-18R), IL-18 alters their activity. IL-18R, which is expressed on the surface of sensory neurons and glial cells, activates several signaling pathways that regulate cellular function (9, 56).

PHN is primarily characterized by neuropathic pain, which is a multifactorial process involving numerous cellular and molecular mechanisms. Glial cell activation, immune inflammation, and alterations in neuroplasticity within the dorsal horn of the spinal cord play a critical role in the manifestation of neuropathic pain (57). The dorsal horn of the spinal cord is a vital center for the transmission and modulation of pain signals. In the context of neuropathic pain, changes in neuroplasticity in the dorsal horn result in the amplification of pain signals and decreased pain thresholds, contributing to the persistence and development of neuropathic pain (58, 59). Dorsal root ganglion (DRG) neurons are responsible for the regulation of pain by IL-18. Several studies have reported that IL-18 expression increases in DRG neurons following nerve injury or inflammation, indicating its potential role in pain sensitization (22, 60, 61). Furthermore, IL-18 regulates various ion channels and receptors associated with pain signaling in DRG neurons (21). Blocking IL-18 signaling has been found to inhibit glial cell hyperactivity and subsequent activation of Ca2+-dependent signaling (36).

IL-18 plays a significant role in the regulation of glial cell activity within the DRG. Astrocytes are situated in close proximity to DRG neurons and serve a critical function in maintaining DRG microenvironmental homeostasis (62). Previous research has demonstrated that IL-18 facilitates microglia and astrocyte interactions, resulting in the release of pro-inflammatory cytokines and the modulation of neuronal activity in the DRG (9). Glial cells, such as microglia and astrocytes, provide essential structural and functional support to neurons. Upon neural injury, these cells are activated and release pro-inflammatory cytokines, chemokines, and other signaling molecules, which stimulate the amplification of pain signals and the recruitment of immune cells to the injury site (63). In PHN, it is suggested that glial cell activation is linked to the maintenance of neuropathic pain (64). IL-18-mediated microglia/astrocyte interactions in the spinal cord are crucial for the development of tactile hypersensitivity symptoms in neuropathic pain (9). Several studies have also demonstrated that spinal cord microglia contribute to the development of inflammation and neuropathic pain through the production of IL-18 (22, 65). Additionally, oligodendrocytes have been shown to play a role in the development of neuroinflammation by producing IL-18 (66).

Immune-mediated inflammation is a key player in the development of neuropathic pain. Following nerve injury, immune cells, such as T cells, B cells, and macrophages, infiltrate the damaged tissue and release pro-inflammatory cytokines and chemokines, which amplify pain signals and promote glial cell activation (67). In PHN, the immune response to the HZ virus is believed to be one of the mechanisms contributing to the development and persistence of neuropathic pain. Animal studies have shown that blocking specific immune molecules, such as interleukin-18, reduces pain sensitivity and improves pain-related behavior (19, 25). NLRP3 inflammatory vesicles are involved in various inflammatory responses, such as those seen in Alzheimer’s disease, diabetes, and neurological injury. High NLRP3 expression can facilitate the secretion of IL-18, which mediates cellular scorching, leading to cell death and playing a crucial role in the development of neurological injury and inflammation-related diseases (35). Moreover, IL-18 activates the inflammasome complex and triggers the production of cytokines, which act on neighboring cells and further amplify the immune response (68). These findings suggest that IL-18 may play a significant role in sensory neuron function and in the pathogenesis of inflammatory and neurological diseases.

The current study has several limitations that should be considered. One limitation is the relatively small sample size of PHN cases in the GWAS database (n=144), which may limit the statistical power of our MR analysis. Secondly, the IL-18 and PHN datasets used in this study were derived from individuals of European origin, and thus the generalizability of our findings to other populations needs to be validated using local GWAS data. For instance, the high prevalence of PHN in China also presents a significant societal burden (69), but our results cannot be extrapolated to the Chinese population until further investigation is conducted. In addition, confidence intervals for MR analysis are relatively wide, reflecting the uncertainty in estimating causal effects. Therefore, we emphasize the need for caution when considering causal relationships inferred from our MR results. Furthermore, due to the lack of information on PHN severity or specific symptoms such as allodynia and nociceptive hypersensitivity, subgroup analysis could not be performed in this study.

## Conclusion

In this study, we conducted bidirectional MR analysis to evaluate the causal effect of IL-18 protein levels on the occurrence of PHN. Our results suggests evidence for a causal relationship between IL-18 protein levels increasing and the occurrence of PHN. However, we did not find evidence supporting a causal relationship between the occurrence of PHN and IL-18 protein levels. These findings offer new insights into identifying and protecting populations at risk of developing PHN and may aid in the development of novel prevention and treatment approaches for PHN.

## Data availability statement

The original contributions presented in the study are included in the article/**Supplementary Material**. Further inquiries can be directed to the corresponding author.

## Author contributions

XL: writing the article, analysis and interpretation. YF: data mining, conception and design, writing the article, critical revision of the article. All authors contributed to the article and approved the submitted version.

## Supplementary material

Supplementary Figure 1 The reverse MR using PHN risk as exposure and IL-18 protein levels (ebi-a-GCST90012024) increasing as outcome. **(A)** Display of the forest plot for the single SNP analysis of PHN risk on IL-18 protein levels. The x-axis shows the MR effect size for PHN risk on IL-18 protein levels, while the y-axis illustrates the analysis for each of the SNPs. **(B)** Presentation of the leave-one-out sensitivity analysis for the effect of PHN SNPs on IL-18 protein levels in the context of MR. The x-axis displays the MR leave-one-out sensitivity analysis for PHN risk on IL-18 protein levels, while the y-axis depicts the analysis for the effect of leaving out individual SNPs on IL-18 protein levels. MR, Mendelian randomization; SNP, single‐nucleotide polymorphism; PHN, Postherpetic neuralgia; IL, Interleukin.

Supplementary Figure 2 The reverse MR using PHN risk as exposure and IL-18 protein levels (prot-b-21) increasing as outcome. **(A)** Display of the forest plot for the single SNP analysis of PHN risk on IL-18 protein levels. The x-axis shows the MR effect size for PHN risk on IL-18 protein levels, while the y-axis illustrates the analysis for each of the SNPs. **(B)** Presentation of the leave-one-out sensitivity analysis for the effect of PHN SNPs on IL-18 protein levels in the context of MR. The x-axis displays the MR leave-one-out sensitivity analysis for PHN risk on IL-18 protein levels, while the y-axis depicts the analysis for the effect of leaving out individual SNPs on IL-18 protein levels. MR, Mendelian randomization; SNP, single‐nucleotide polymorphism; PHN, Postherpetic neuralgia; IL, Interleukin.
