# Supplementary figures and images for: Bidirectional two-sample Mendelian randomization analysis reveals a causal effect of interleukin-18 levels on postherpetic neuralgia risk
Source: Front Immunol. 2023 May 25;14:1183378. doi: 10.3389/fimmu.2023.1183378 (PMC10247971; doi:10.3389/fimmu.2023.1183378)

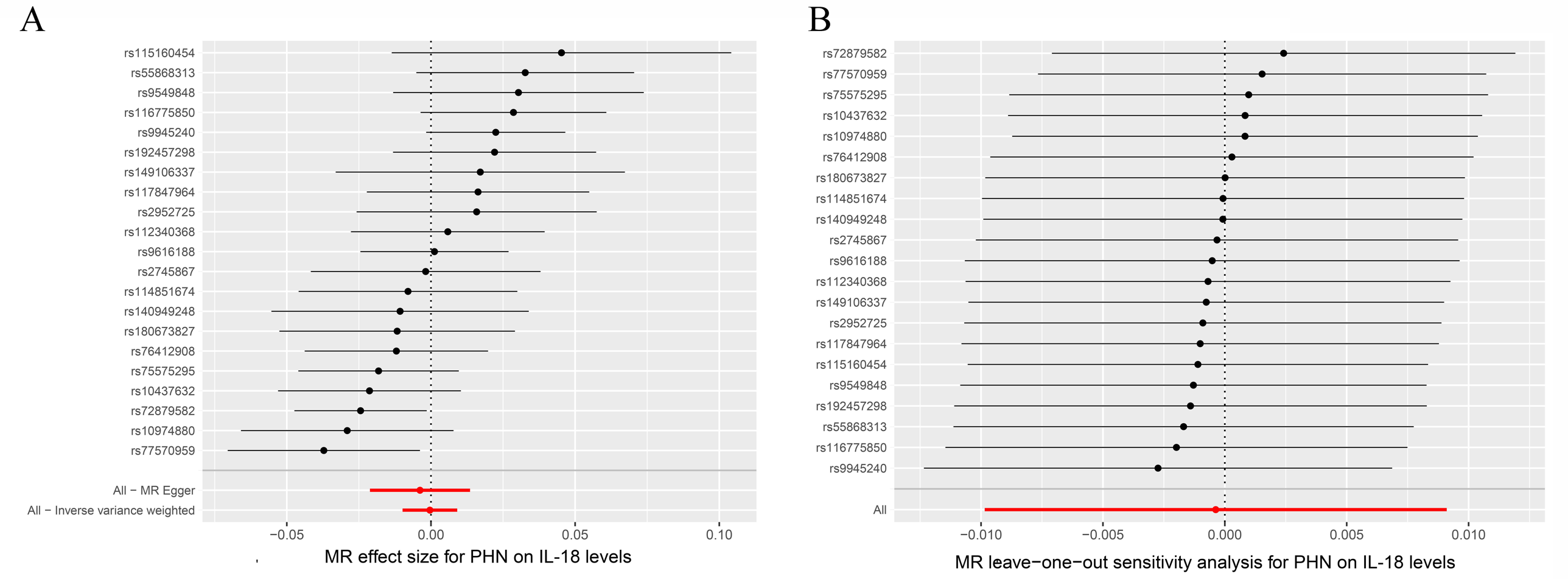

Supplement: Supplementary Figure 1 — The reverse MR using PHN risk as exposure and IL-18 protein levels (ebi-a-GCST90012024) increasing as outcome. (A) Display of the forest plot for the single SNP analysis of PHN risk on IL-18 protein levels. The x-axis shows the MR effect size for PHN risk on IL-18 protein levels, while the y-axis illustrates the analysis for each of the SNPs. (B) Presentation of the leave-one-out sensitivity analysis for the effect of PHN SNPs on IL-18 protein levels in the context of MR. The x-axis displays the MR leave-one-out sensitivity analysis for PHN risk on IL-18 protein levels, while the y-axis depicts the analysis for the effect of leaving out individual SNPs on IL-18 protein levels. MR, Mendelian randomization; SNP, single‐nucleotide polymorphism; PHN, Postherpetic neuralgia; IL, Interleukin. [file Image_1.jpeg]

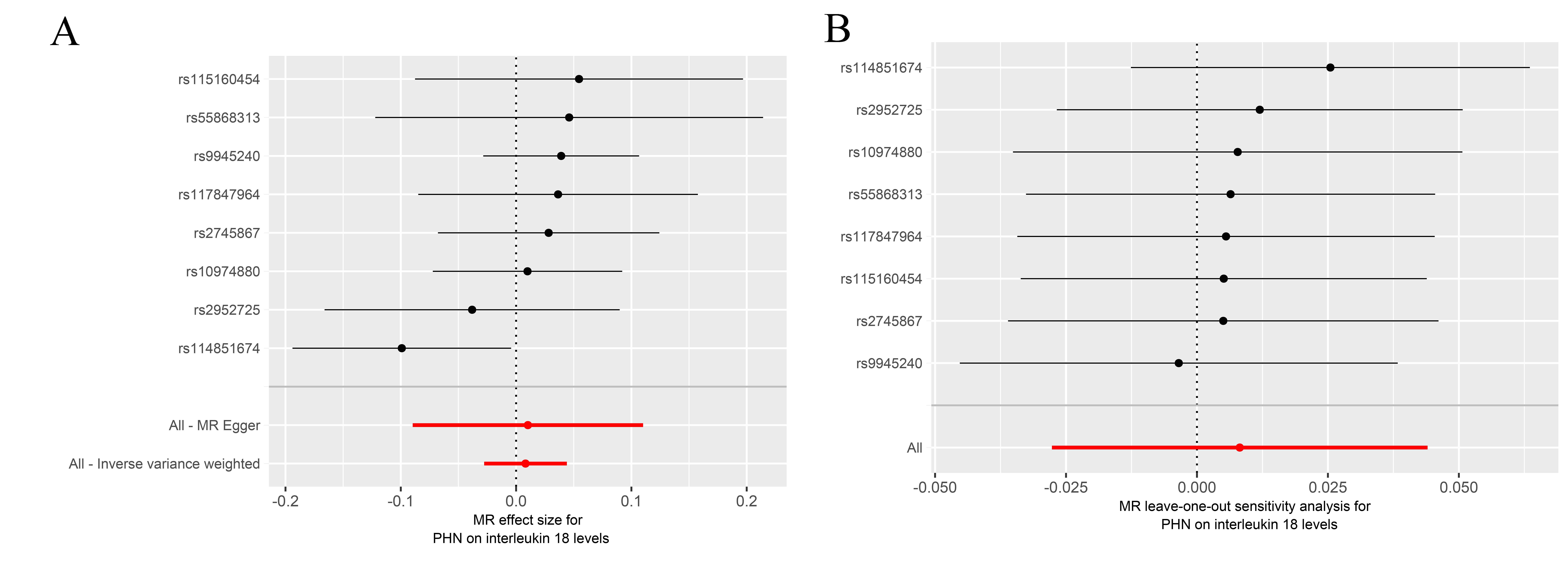

Supplement: Supplementary Figure 2 — The reverse MR using PHN risk as exposure and IL-18 protein levels (prot-b-21) increasing as outcome. (A) Display of the forest plot for the single SNP analysis of PHN risk on IL-18 protein levels. The x-axis shows the MR effect size for PHN risk on IL-18 protein levels, while the y-axis illustrates the analysis for each of the SNPs. (B) Presentation of the leave-one-out sensitivity analysis for the effect of PHN SNPs on IL-18 protein levels in the context of MR. The x-axis displays the MR leave-one-out sensitivity analysis for PHN risk on IL-18 protein levels, while the y-axis depicts the analysis for the effect of leaving out individual SNPs on IL-18 protein levels. MR, Mendelian randomization; SNP, single‐nucleotide polymorphism; PHN, Postherpetic neuralgia; IL, Interleukin. [file Image_2.jpeg]
